# Reliability of the digital functionally generated path technique for assessing occlusal interferences and adjusting CAD-CAM zirconia crowns: an in vivo study

**DOI:** 10.1186/s12903-024-05202-9

**Published:** 2024-11-22

**Authors:** Sherine Anwar Saad, Yousreya Atteya Shalaby, Amir Shoukry Azer

**Affiliations:** Fixed Prosthodontics Department, Faculty of Dentistry, Alexandria University, Roushdy, Sidi Gaber, Alexandria, Egypt

**Keywords:** CAD-CAM, Dynamic occlusion, Intraoral scanner, Occlusal adjustment, Occlusal interferences

## Abstract

**Background:**

Designing the occlusal surface of a prosthesis to ensure optimal eccentric occlusion is challenging without precisely replicating the patient’s mandibular movements. During the CAD-CAM prosthesis delivery process, clinicians often need to make adjustments to the prosthesis to avoid occlusal interferences that may occur during lateral excursions. Recently, there have been developments in the field of mandibular motion tracking using optical devices. These approaches seek to incorporate an individual’s functional movement into the research field of occlusal morphology.

**Aim:**

This study aimed to assess the accuracy of digitally replicating mandibular movements to identify and correct occlusal interferences in monolithic CAD-CAM zirconia crowns.

**Methods:**

An intraoral scanner (IOS) was used to capture complete arch maxillary and mandibular teeth and record buccal and lateral interocclusal records of maxillary first premolar abutment teeth of thirteen participants. For each patient, two monolithic zirconia crowns were fabricated following the standard digital workflow. The crowns were categorized based on the virtual method used for adjusting occlusal interferences into two groups: Group I, where occlusal interferences in CAD-CAM zirconia crowns were adjusted using buccal interocclusal records, and Group II, where adjustments were made using both buccal and lateral interocclusal records. After crown fabrication following the manufacturer’s instructions, occlusion was analyzed using an electronic pressure analyzer. The mean, standard deviation and median values of the recorded data were measured. Paired t test and Wilcoxon Sign Rank test were executed for analyzing differences between groups (p value ≤ 0.05).

**Results:**

Group I recorded higher maximum pressure at lateral mandibular movement with mean ± standard deviation value of 26.00 ± 4.95% than Group II with 20.62 ± 3.38%. Regarding pressure recorded at maximum intercuspation (MI) Group I showed higher results; 8.08 ± 1.50% compared to Group II with 7.23 ± 1.59% mean ± standard deviation value. The average value of crown volume for Group I was (160.36 ± 15.94) mm^3^, while for Group II was (157.63 ± 14.45) mm^3^.

**Conclusions:**

The digital functionally generated path technique allows for identifying occlusal interferences and modifying CAD-CAM zirconia crown designs.

## Background

The dental prosthesis within the oral cavity must be in harmony with the natural functions and anatomy of the stomatognathic system. A widely accepted principle in occlusal design emphasizes that dental prothesis must be as similar as possible to the natural dental anatomy and establish a balanced interaction with the neighboring and opposing structures [1]. Furthermore, dental prostheses must ensure optimal function at maximum intercuspation (MI) and should not disrupt mastication and articulation during eccentric movements. However, reproducing the structural and functional occlusal surface characteristics of premolars and molars in restorations remains challenging in dentistry as a result of potential discrepancies that may arise during clinical and laboratory procedures [2].

In the traditional approach, the quality of the final prothesis is linked to the expertise of the clinician and the laboratory technician, as well as the properties of the materials used [3]. In contrast, a completely digitalized workflow employing computer-aided design and computer-aided manufacturing (CAD-CAM) technology utilizes intraoral scanners (IOSs) to offer accurate three-dimensional (3D) information about the abutment tooth, neighboring teeth, and antagonist teeth. This information is then utilized for the design of the prothesis [4].

The CAD software program typically creates the occlusal surface of crowns using a standard design, which often requires additional adjustments to the occlusion. To minimize the need for manual adjustments during dental procedures, CAD software often incorporates a virtual articulator module into its digital workflow. Nevertheless, due to the inherent similarity between the virtual articulator and the conventional mechanical articulator, designing a fully functional occlusion in the digital workflow is a challenging task. Currently, crown restorations in CAD software are primarily designed using the expertise of technicians [5].

As a result of advances in CAD software programs, algorithms are currently used to adapt the biting surface to both static and dynamic occlusions, including the biogeneric tooth model [6]. Data are obtained from the shape and configuration of the remaining teeth and how they come together using a mathematical algorithm. This technique produces a genuine occlusal surface that seamlessly matches the neighboring teeth [7]. The mirroring technique permits the operator to duplicate the tooth on the opposite side and establish a replica of the preparation [8]. The biogeneric copy design mode can accurately reproduce the dental structure of already existing teeth, diagnostic wax patterns, and interim designs [9].

Recently, innovative approaches involving the tracking of mandibular motion through optical devices have emerged. One such method, known as Patient Specific Motion (PSM) (3Shape A/S in Copenhagen, Denmark) that enables the recording of an individual’s dynamic occlusion during lateral movement using an Intraoral Scanner. This technique accumulates sequential mandibular motion data within the individual’s oral cavity utilizing IOS. The digitally recorded dataset has a twofold function: it allows for a visualization of mandibular movement and aids in adjusting occlusal errors in CAD software while designing the occlusion. By replicating the dynamic functional movement in CAD software, the PSM may successfully reduce occlusal interference during eccentric movements [10].

Employing digital scanning may maximize process efficiency, enhance patient satisfaction, decrease treatment durations, streamline laboratory production and enhance communication between the dentist and the dental laboratory technician [11]. Also, crowns made with a digitalized workflow have shown to have better marginal adaptation, proximal fit, occlusal surface contacts and crown anatomy [11, 12]. In the digital workflow, it is expected that individuals with limited expertise, including both dentists and technicians, possess the ability to produce prothesis of comparable standards to those created by experienced individuals [13, 14].

Digital occlusal assessment tools are utilized to precisely capture the precise position of occlusal contacts and the corresponding force exerted on the occlusal contacts at various intervals. This data is highly valuable for conducting occlusal analysis in the fields of prosthodontics and maxillofacial prosthetics [15]. According to the recordings, the software of the digital occlusal analysis systems executes a series of computations to analyze the forces applied to individual teeth, ascertain the center of contact for these forces and assess the equilibrium between right and left arch sides.

The most extensively researched device [16] to enter the market was the first (T-Scan; Tekscan Inc.), which has been evaluated for its precision in identifying the location of occlusal contacts and measuring contact force magnitude [17, 18]. Recently, a newer digital occlusal analyzer (Occlusense; Dr. Jean Bausch GmbH & Co. KG) has been introduced, offering a lower-cost alternative. This device includes a sensor equipped with articulating paper that directly marks occlusal contacts on teeth. The advanced pressure sensors in Occlusense (60 microns thick) can capture masticatory forces across 256 pressure levels. Its thin and flexible design enables recording of both static and dynamic occlusions. When the Occlusense sensor is attached to the handheld device, the device becomes ready for the digital occlusal analysis test. The handheld then wirelessly transmits the recorded data to the Occlusense iPad application which saves and visually presents the raw data on the iPad screen. The Occlusense handle is capable of capturing incremental digital occlusal data at a rate of 0.056 s per frame. Comparative studies have indicated reasonable similarity in results between the two devices [19].

The aim of this clinical investigation was to assess the accuracy of digitally replicating mandibular movements to identify and correct occlusal interferences in designed and milled monolithic CAD-CAM zirconia crowns using a digital occlusal analyzer. The null hypothesis posited that there would be no significant difference in the detection and elimination of occlusal interferences between CAD-CAM zirconia crowns designed using both buccal and lateral bite records versus those designed using buccal bite records alone.

## Materials and methods

The Research Ethics Committee of the Faculty of Dentistry at Alexandria University in Egypt granted official authorization for this clinical study (0266-07/2021) and the clinical trial number for the present study was (NCT06272474). Each participant willingly granted their written consent. For the participants to be enrolled in the study, they had to meet specific requirements. The criteria included a maxillary first premolar that had undergone successful endodontic treatment, class I occlusion, canine guided occlusion, good oral hygiene without indications of periapical lesions or periodontal pathology and having natural teeth as opposing dentition. The study did not include participants with occlusal interferences, increased tooth mobility, or parafunctional habits. Thirteen participants (8 women and 5 men aged from 18 to 25 years) from Faculty of Dentistry, Alexandria University were recruited. Sample size was estimated assuming 5% alpha error and 80% study power. According to Lee et al. [20], estimates for deviation out of tolerance level are considered as indication for occlusal errors that require adjustments. The mean ± SD values out of tolerance were 173.1 ± 31.3 and 210.9 ± 48.6 for patient specific motion (buccal and lateral bite) and static occlusion (buccal bite only), respectively [20]. Based on comparison of dependent means, the minimum sample size was calculated to be 13 patients. Sample size was based on Rosner’s method [21] calculated by Gpower 3.0.10.

**Fig. 1:**
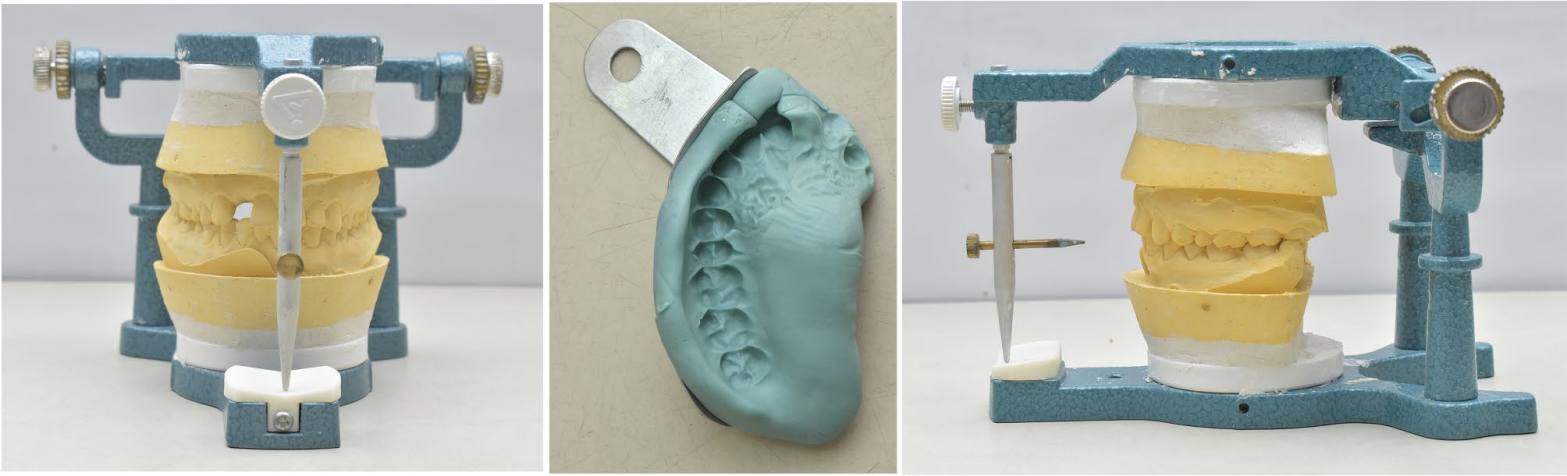
Study casts mounted on mean value articulator and putty index in sectional tray

**Fig. 2:**
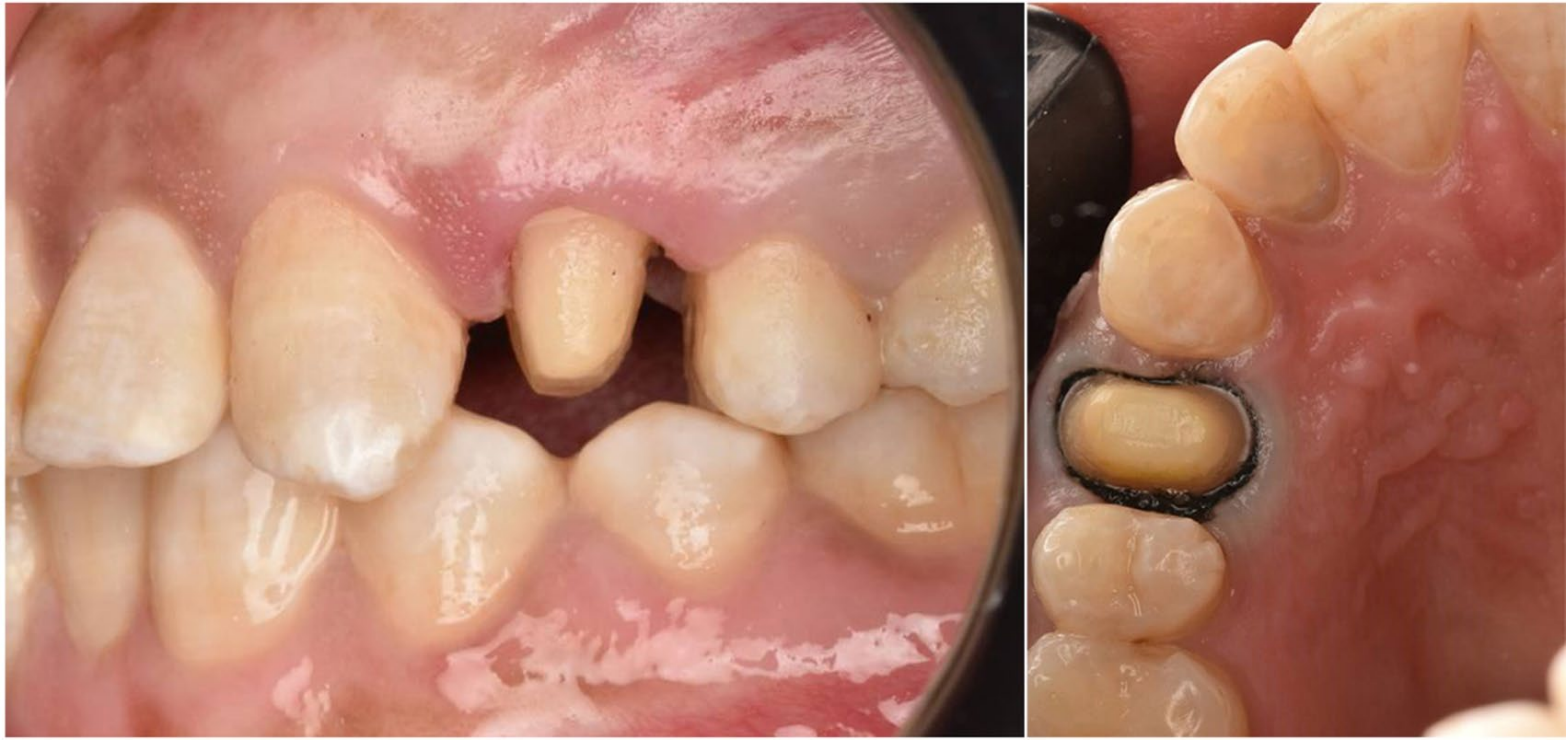
Abutment tooth prepared for all ceramic restoration

Materials used in this study are presented in Table 1. A preoperative periapical radiographic assessment was conducted for each patient. Subsequently, an alginate impression was obtained for each patient using the appropriate size of stainless-steel tray (Generic, perforated trays, OMEGA) [22]. Following the manufacturer’s instructions, each impression was poured with dental stone. The study cast for each patient was mounted on a mean value articulator (Indian articulator, Dentmark), utilizing a wax interocclusal record, to facilitate preoperative examination and the fabrication of provisional crowns (Fig. 1). A condensation silicon putty index was molded over the abutment tooth and adjacent teeth. It was then cut in the middle of abutment tooth using a scalpel, to be then used for assessing the amount of reduction. Following the clinical diagnosis, the abutment teeth underwent preparation for all-ceramic restoration in accordance with the standard preparation guidelines [23]. The procedure involved reduction of the occlusal surface by 1.5 mm, axial reduction with a 1 mm-wide circumferential deep chamfer margin and an equigingival finish line (Fig. 2). All sharp preparation angles were rounded as well. A provisional crown was fabricated for each patient to be temporarily cemented between the visits. Composite based provisional material was applied directly into the silicon putty mold shown in Fig. 1using an auto mixing gun (3 M ESPE, St. Paul, MN, USA). The putty mold was then seated on the abutment tooth to create the provisional crown, which was then finished and polished prior to cementation.

After calibration as per the manufacturer’s guidelines using a calibration device, Medit i500 IOS (Medit Corp, Seoul, Korea. version 2.2) was used to capture complete-arch scans and record buccal occlusion using the recommended protocol. The maxillary arch scan started at the posterior left occlusal area, advanced towards the contralateral side using a zig-zag pattern in the anterior teeth region, turned palatal, and ended on the buccal side of the right area. The mandibular arch scan started at the posterior left occlusal region, progressed towards the contralateral side using a zig-zag motion in the anterior teeth, turned lingually toward the left quadrant, and concluded on the buccal side of the right posterior quadrant.

**Fig. 3:**
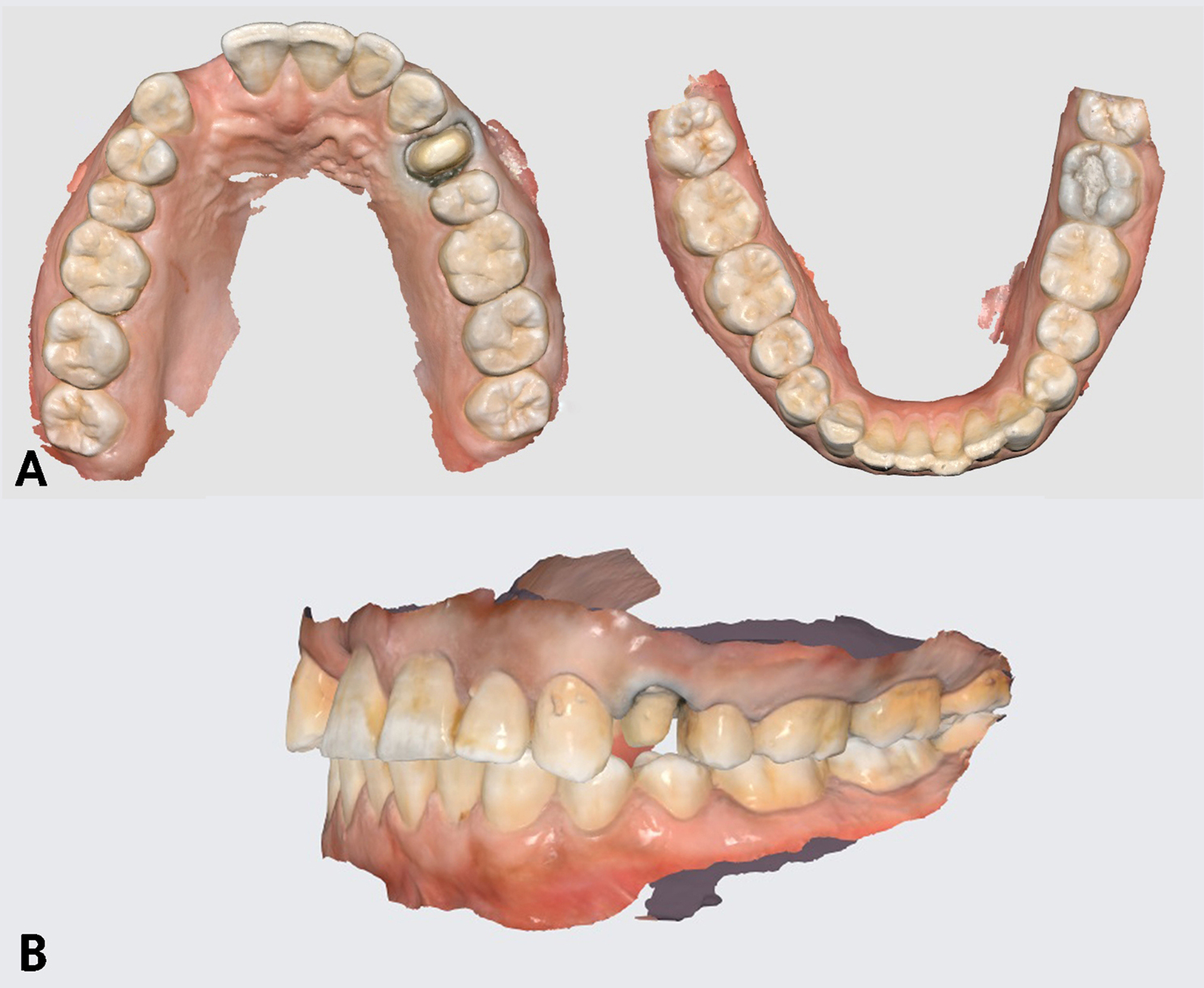
Scanning procedure using IOS (Medit i500 version 2.2). **A**, Complete-arch scans. **B**, Replicating the occlusal state of the digital casts employing the buccal bite registration method (BBR)

Precautions were taken to avoid a strong external light source during the scan. Additionally, ensuring the mirror was clean and free of stains was a prerequisite before each scan. The scanner tip was moved uniformly at a moderate speed to ensure optimal alignment. Using the buccal bite registration method (BBR), the IOS promptly replicated the occlusal state of the digital casts, (Fig. 3A, B). For the recording of lateral mandibular movement, another scan was conducted for each patient. Following the acquisition of aligned maxillary and mandibular scan data and selecting the desired mandibular movement direction icon (working lateral direction). The patient was directed to close at maximum intercuspation (MI), then the patient was instructed to move their mandible laterally while maintaining contact between the maxillary and mandibular teeth until only canine tooth contact occurred. Then, three standard tessellation language (STL) files were exported; maxillary arch, mandibular arch at MIP and lateral mandibular movement recording files.

**Fig. 4:**
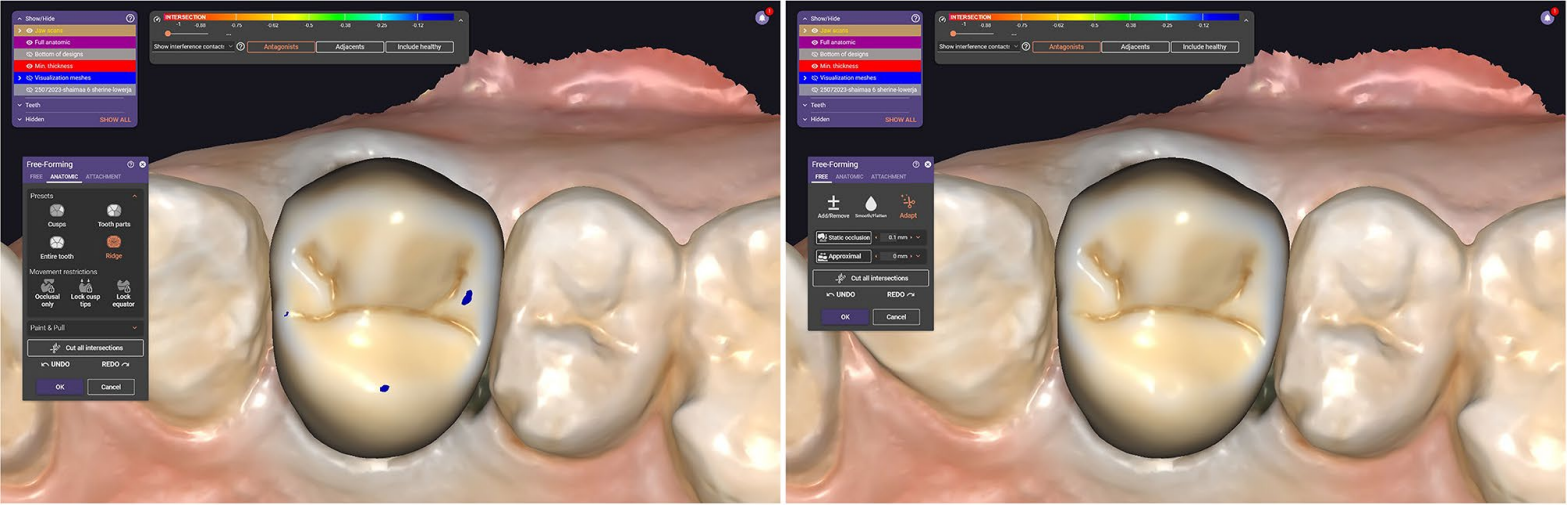
Crown designed using standard digital workflow of CAD software utilizing the MI antagonist scan data. Crown interferences regarding static occlusion eliminated utilizing Exocad cutting tool. Reference crown design. CAD, computer-aided design; MI, maximum intercuspation

**Fig. 5:**
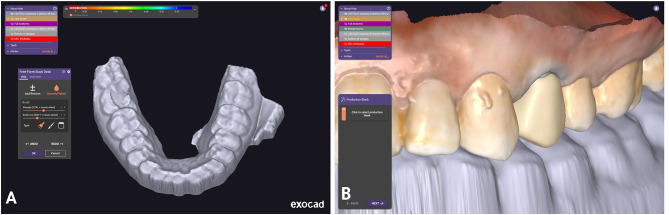
Test crown design. **A**, Antagonist cast scan data related to lateral mandibular excursion. **B**, Maxillary arch with Reference restoration design data and antagonist cast scan data related to lateral mandibular excursion were imported into the CAD software. CAD, computer-aided design

**Fig. 6:**
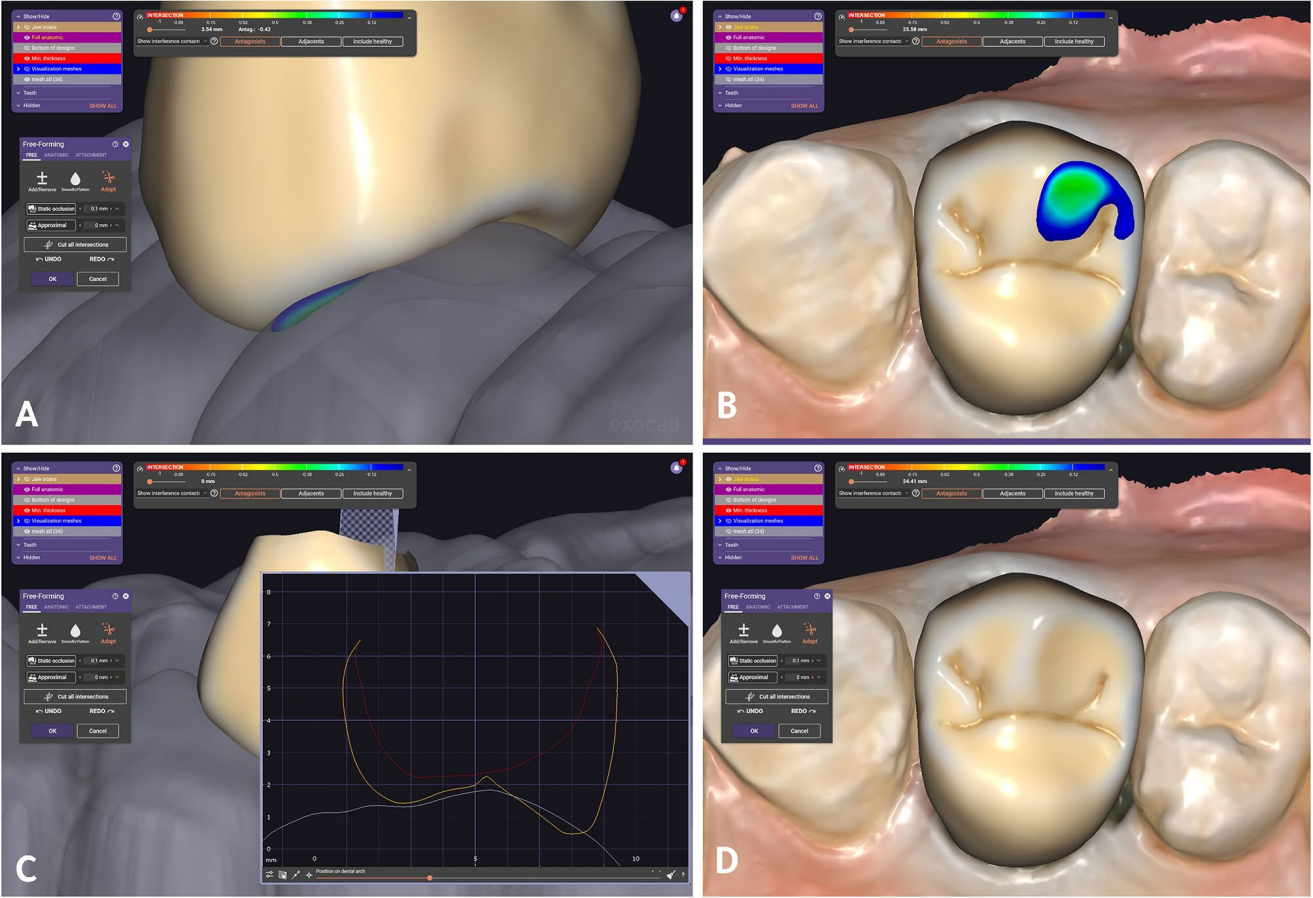
Test crown design **A**, **B**, **C**, Crown interferences regarding dynamic occlusion detected by CAD software. **D**, Interferences removed utilizing Exocad intersection cutting tool. CAD, computer-aided design

The three STL files were imported into the Exocad software (Version I CAD proga. 3.1 Rejeka). The design of the crown contour was accomplished using the maxillary arch scan data and MIP antagonist scan data, employing the Exocad software within a standard digitalized process. The crowns for all patients were designed utilizing the same Exocad tooth library with cement gap set to be 50 microns and Occlusal interferences removed with 0-mm clearance. Subsequently, the Exocad cutting tool was employed to eliminate any crown interferences at static occlusion. Finally, STL file for the definitive crown design considering maximum intercuspation was saved as Group I (Reference crown) (Fig. 4). The antagonist scan data for maximum intercuspation (MI) was then excluded, and subsequently, the maxillary arch with restoration scan data and mandibular cast scan data related to lateral mandibular movement were imported into the CAD software (Fig. 5). Subsequently, the overlapping area between the designed crown data and the antagonist scan data considering the lateral mandibular excursion was confirmed (Fig. 6A, B, C). The identified overlap area was removed utilizing the intersection cutting tool of the software. Subsequently, STL file for the final crown design concerning maximum intercuspation (MI) and lateral mandibular excursion was saved as Group II (Test crown) (Fig. 6D).

**Fig. 7:**
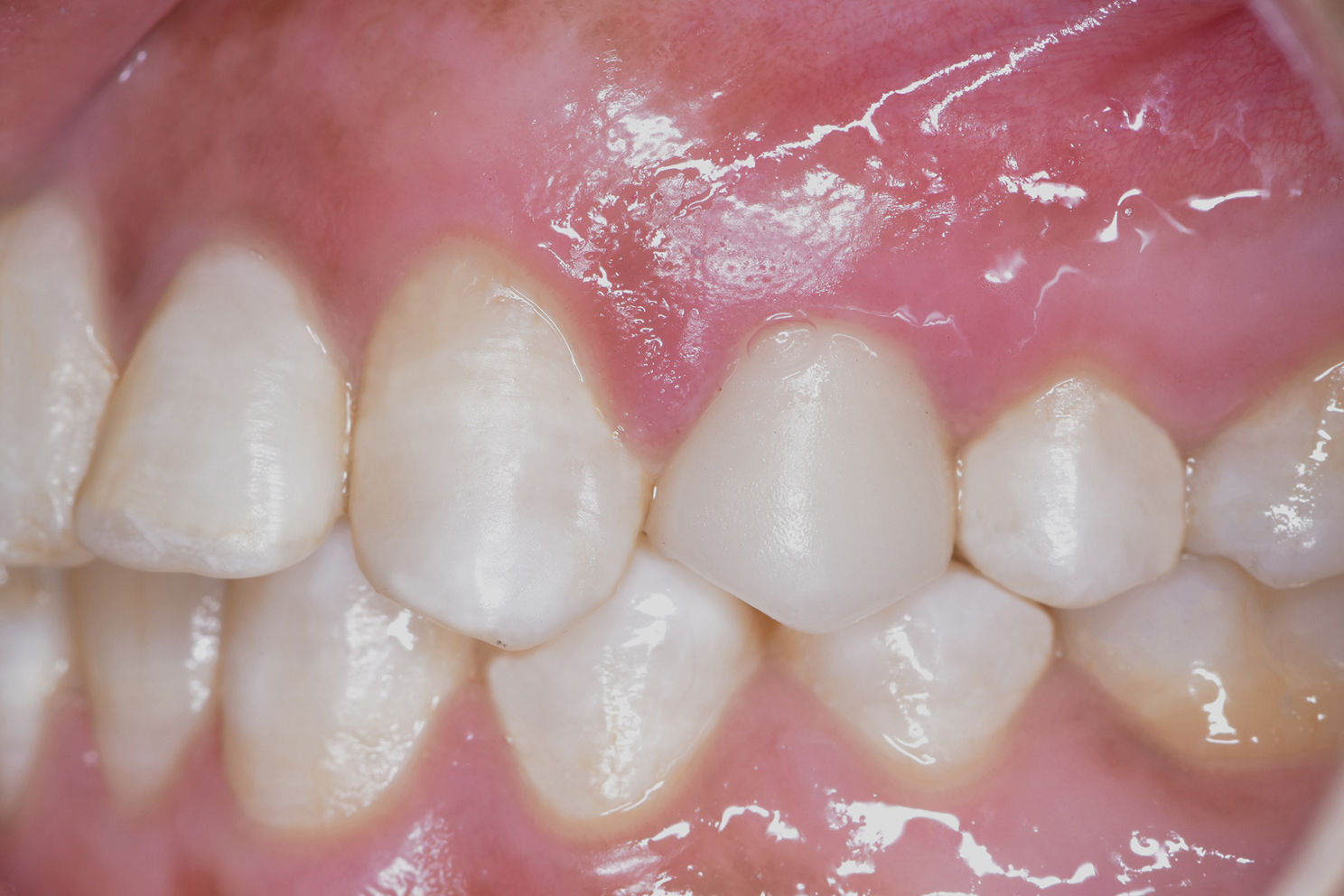
Definitive crown temporarily cemented and set for digital occlusal scheme analysis

Subsequently, the milling and sintering processes for all monolithic zirconia crowns were executed, encompassing two crowns for each patient, in strict accordance with the guidelines provided by the manufacturer. It’s important to note that all procedures were performed by a single operator, all crowns underwent milling from the same zirconia blank (Zenostar, Ivoclar Vivadent AG), utilizing the same CAD-CAM milling machine (Ceramill, Amann Girrbach, Austria) and undergoing the identical sintering procedure. Before the digital occlusal scheme analysis, the reference crown for each patient was temporarily cemented using zinc oxide temporary cement (Fig. 7). Each patient was instructed to bite on a cotton roll placed at the crown’s occlusal surface and at the same time on addition silicon bite registration material placed contralaterally to create an index. A 5-minute interval was left between the temporary cementation and the occlusal analysis process using the occlusense to ensure cement setting. Reference crown was then removed using an excavator tip, the silicon index was replaced at the exact position utilizing cuspal indentations and the same previous steps were repeated for cementation of the test crown. The silicon index aided to maintain a standardized seating pressure for both crowns prior to occlusal analysis.

**Fig. 8:**
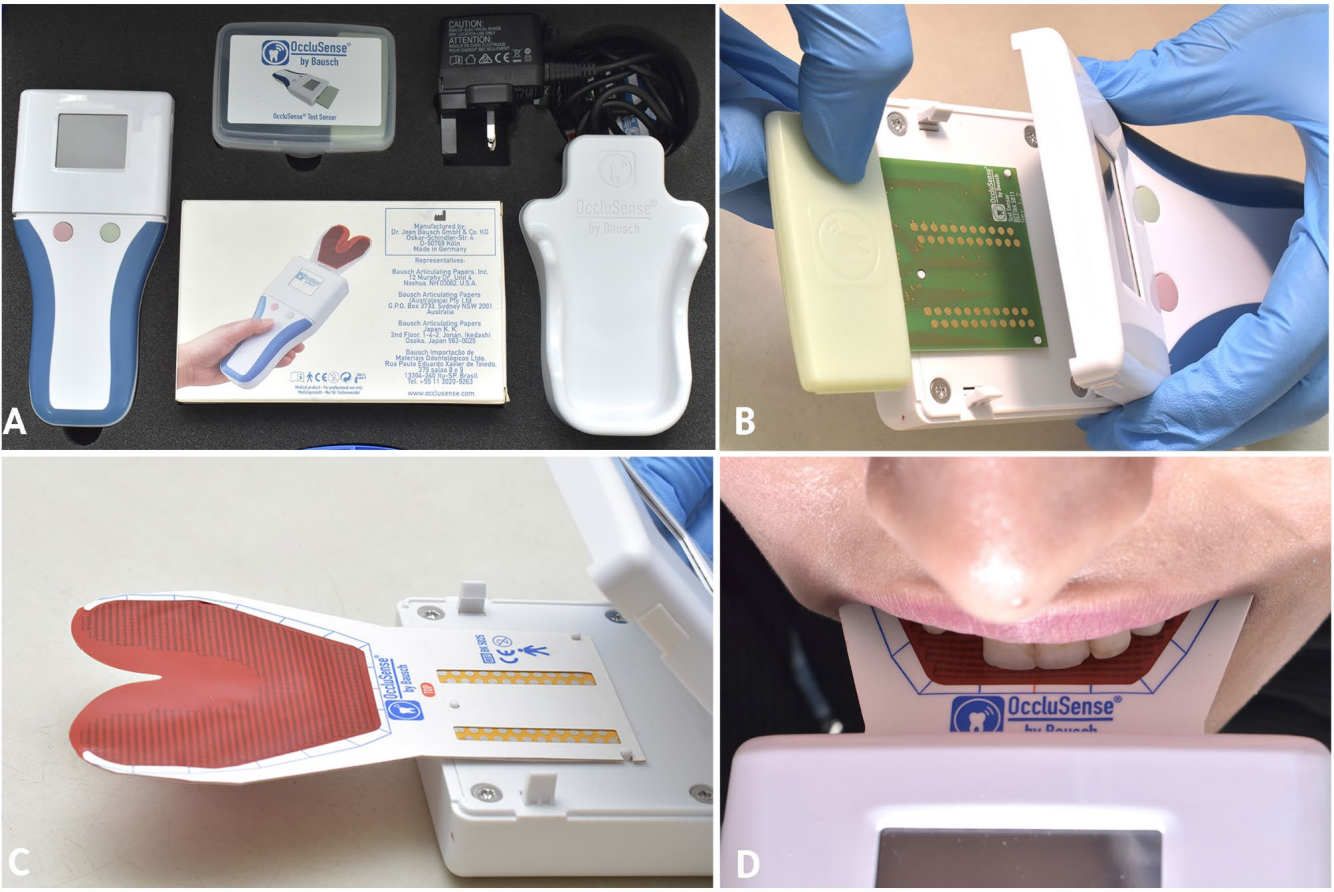
Digital occlusal analyzer. **A**, Components of the occlusence device. **B**, Handle Function Test performed with Test Sensor. **C**, Sensor placed into the handheld guided by pins. **D**, The red line on the sensor aligned with the dental midline

For digital analysis of the occlusal scheme, all clinical workflows were performed with participants positioned in a dental chair at a 90-degree angle, ensuring their Frankfort plane was parallel to the floor. The Occlusence was utilized to gather static and dynamic occlusion data for the two designated crowns of every participant (Fig. 8A). Before using the Occlusense System for the first time, the Occlusense application was installed on the ipad. And prior to initial operation of the Occlusense System or after reset to factory default, a network configuration of the handheld and the associated pairing with the iPad was performed as per manufacturer instructions. Furthermore, after connecting the Occlusense handle to the iPad, a Handle Function Test was performed using a Test Sensor before each clinical session (Fig. 8B). Subsequently, utilizing the handheld device’s pins, the disposable sensor was strategically placed in the manner depicted in Fig. 8C. The recording settings were set to 50 Hz over a 4-second duration, adhering to the manufacturer’s guidelines.

For each participant, four recordings were registered. After temporarily cementing the reference crown, participants were guided to close at maximum intercuspation on the color-coated foil of the sensor, aligning the red line located at the top middle of the sensor with their dental midline (Fig. 8D). Subsequently, the participant was directed to execute lateral movement of the mandible (towards the working side) while maintaining occlusal contact. The maximum pressure during lateral mandibular movement was identified by reviewing the video recording of the occlusal analysis. The previous recordings were repeated for the test crown. A 2-minute rest period was implemented between occlusal records to prevent muscle fatigue. Furthermore, a blind protocol was used in the present study, participants were not informed which crown was under clinical investigation.

**Fig. 9:**
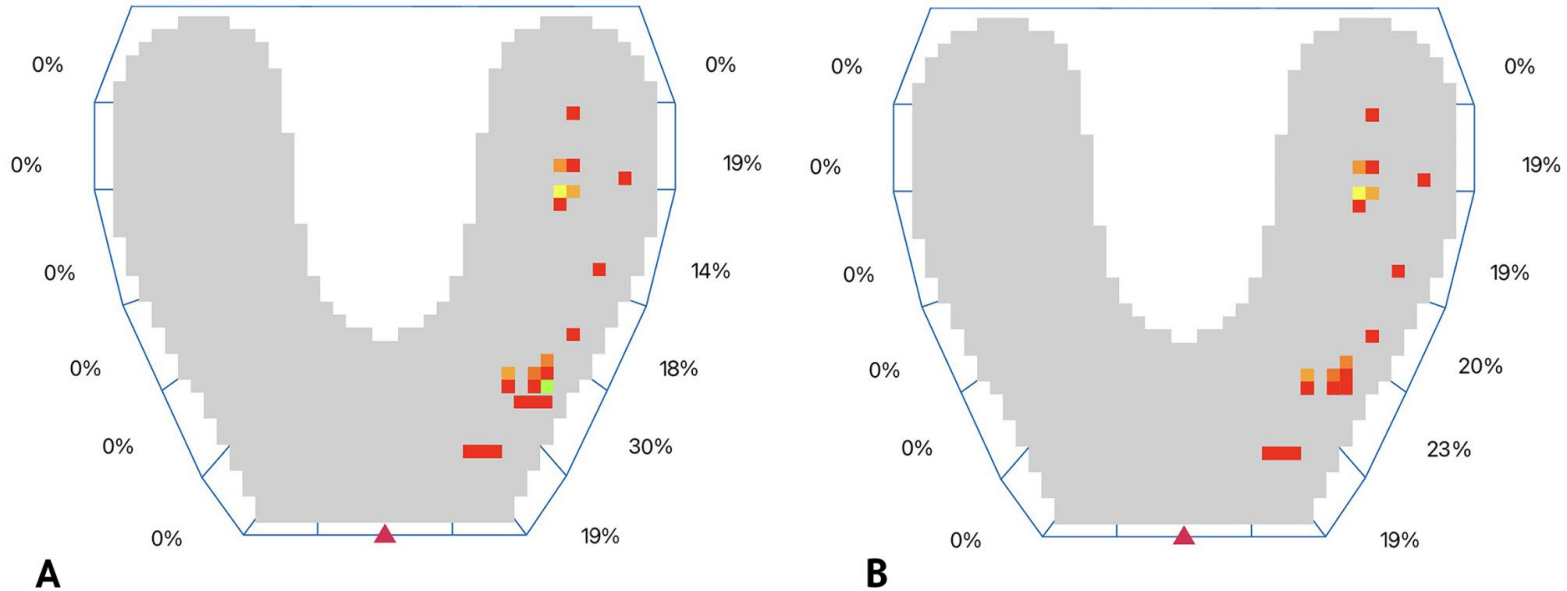
Digital occlusal analysis at lateral mandibular excursion. **A**, Reference crown. **B**, Test crown. Color map showing cusp steepness

**Fig. 10:**
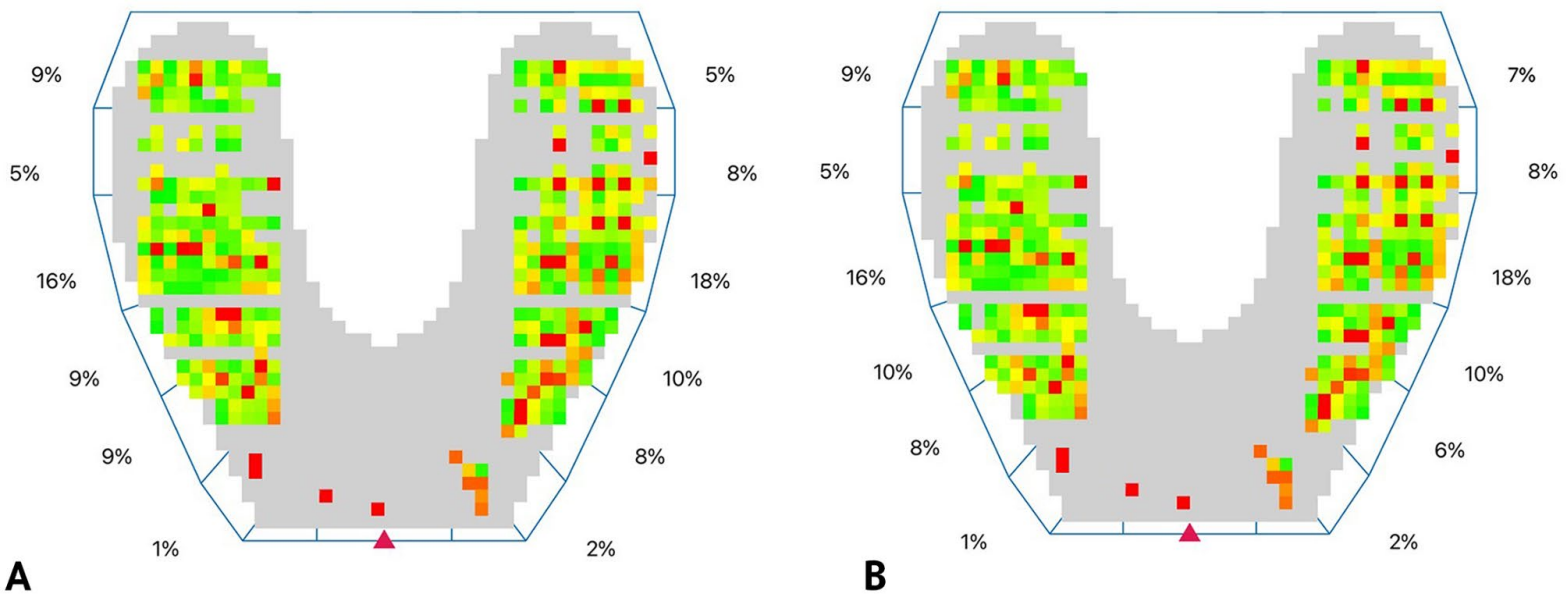
Digital occlusal analysis at maximum intercuspation. **A**, Reference crown. **B**, Test crown. Color map showing cusp steepness

All data was automatically saved on the iPad application. Static and dynamic occlusal analyses were performed on the reference crown and the test crown, respectively (Figs. 9 and 10).

**Fig. 11:**
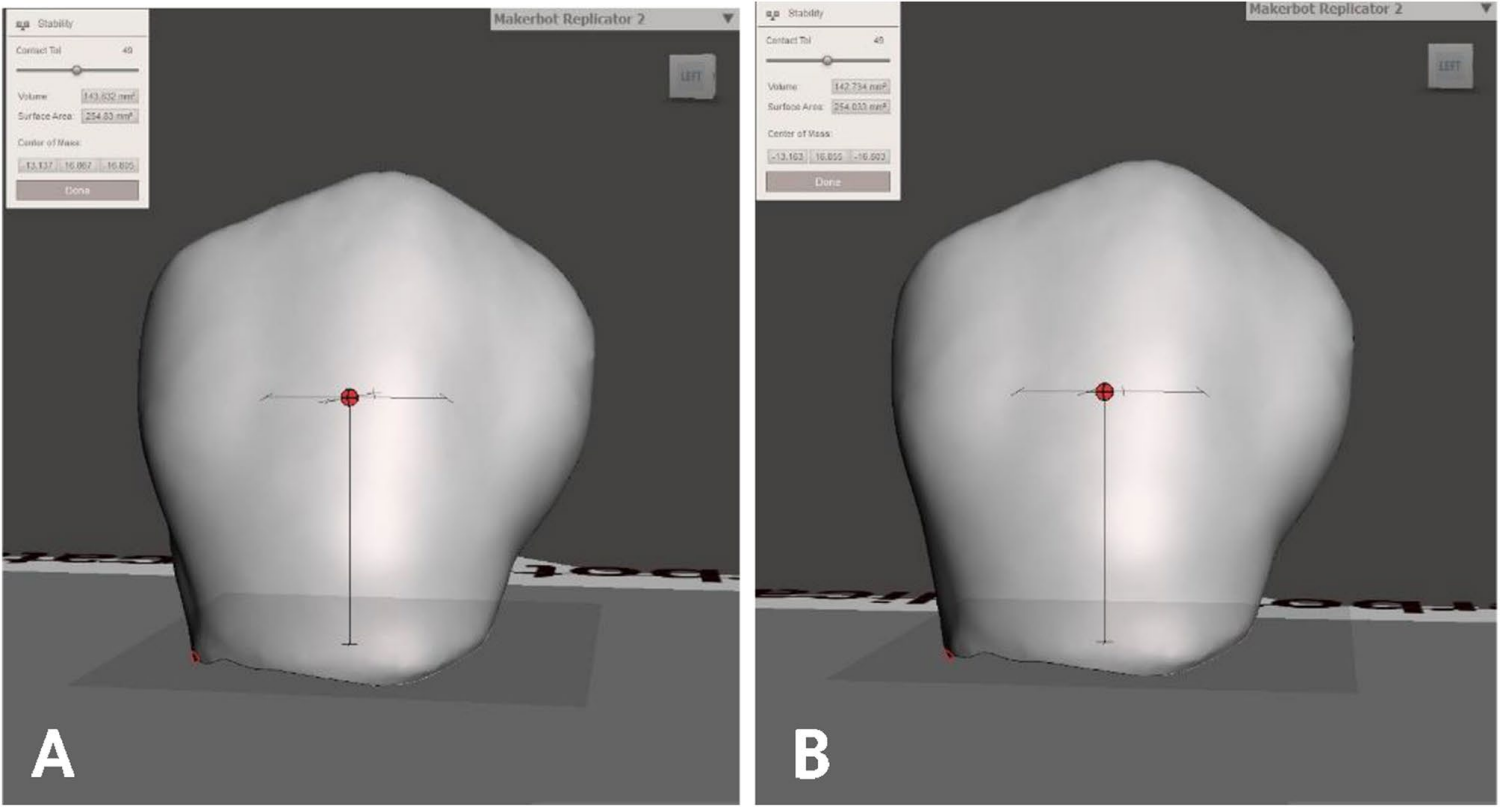
Crown volume calculation using Meshmixer software. **A**, Reference crown. **B**, Test crown

All standard tessellation language (STL) files were brought into Meshmixer software (MeshMixer; Autodesk, San Rafael, USA) to determine the crown surface area (mm^2^) and crown volume (mm^3^) for both the reference and test crowns of each participant (Fig. 11). The crown volume was computed by multiplying the crown surface area by the crown height measured from the center of mass of each crown, as per the software’s calculation [24].

Normality was verified using Shaprio Wilk test and Q-Q plots. Paired t test was used for statistical analysis depending on the normality. All tests were two tailed and the significance level was set at p value ≤ 0.05. Data were analyzed using IBM SPSS, version 23 for windows, Armonk, NY, USA.

**Table 1:** Materials used in this study

Material	Manufacturer	Lot number
Alginate impression	New Kromopan, Schultz Science Dental Product, Jerman	15,038,011,062
Dental stone	Silky Rock (SR), Whip Mix Corporation, Louisville, USA	230,391
Condensation silicon	Zeta plus Zhermack SpA, Badia Polesine (RO), Italy	10,045,021
Composite based provisional material	Charm temp Dent Kist, Inc. Korea	110,047
Temporary cement	Cavex Holland BV, Haarlem, Netherlands	2031CJ
Addition silicon bite registration material	Dreve Dentamid GmbH, Unna, Germany	10,057,613

## Results

**Fig. 12:**
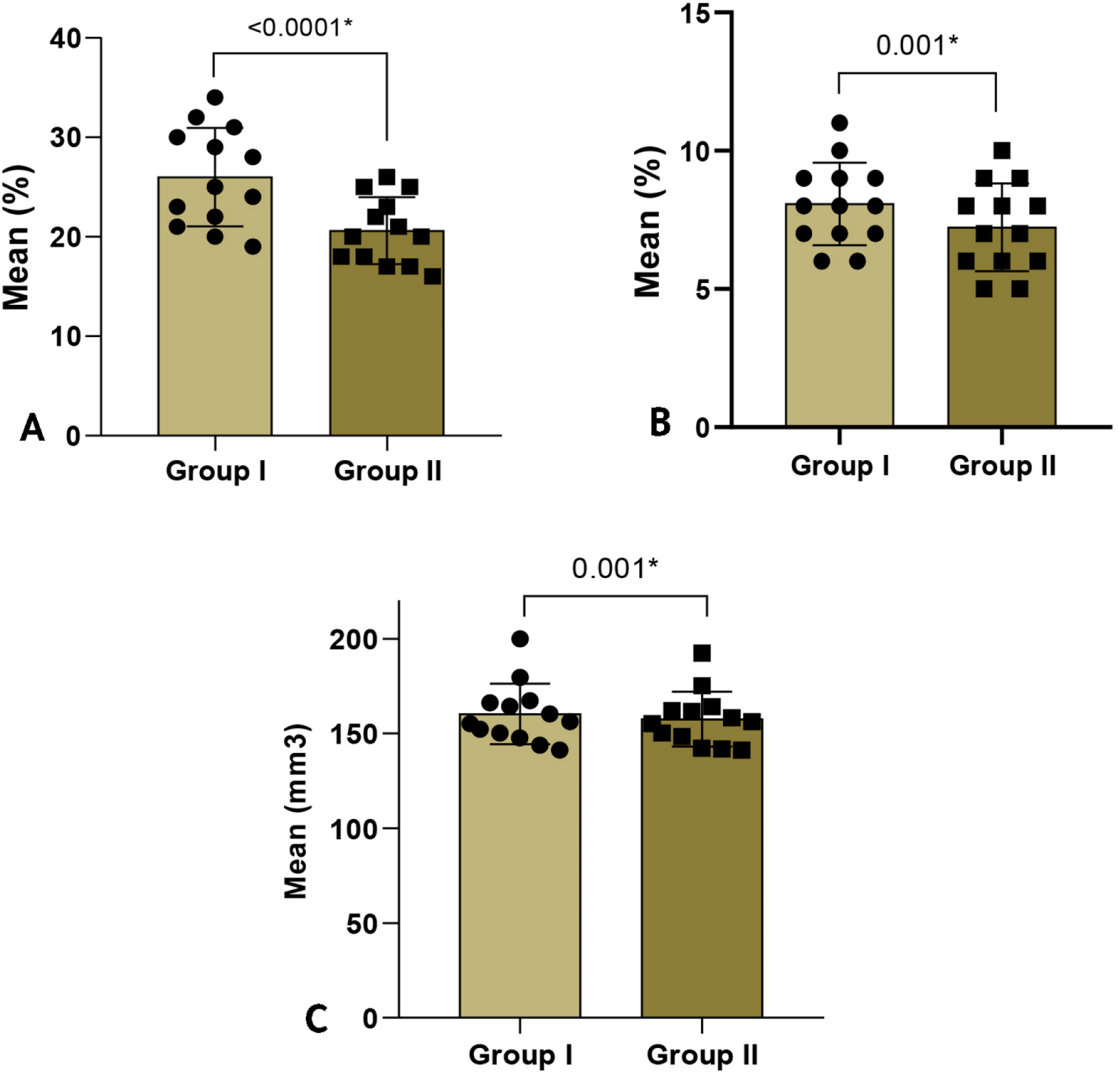
Comparison between Group I [Reference crowns] and Group II [Test crowns] **A**, Regarding maximum pressure recorded during lateral mandibular movement [working side]. **B**, pressure recorded at maximum intercuspation. **C**, regarding crown volume

The mean value of maximum pressure recorded during lateral mandibular movement for Group I (Reference crowns) showed higher significance than Group II (Test crowns) with a p-value < 0.0001, as confirmed by the Paired t-test (Table 2) (Fig. 12A).

**Table 2:** Comparison between groups regarding maximum pressure recorded during lateral mandibular movement [working side]

Patient number	Group I (Reference) *n* = 13	Group II (Test) *n* = 13
Mean ± SD (%)	26.00 ± 4.95	20.62 ± 3.38
95% CI	23.01, 28.99	18.57, 22.66
t Test (*p* value)	5.830 (< 0.0001*)	

Group I, CAD-CAM zirconia crowns adjusted using buccal interocclusal record; Group II, CAD-CAM zirconia crowns adjusted using buccal and lateral interocclusal records. *Statistically significant difference at p value ≤ 0.05, t Test: Paired t test

A comparison of the pressure recorded at maximum intercuspation between Group I and Group II is displayed in Table 3. By using a Paired t-test, it was found that Group I crowns had a significantly higher mean value of pressure recorded at MI than Group II crowns (p-value = 0.001) (Fig. 12B).

**Table 3:** Comparison between groups regarding pressure recorded at maximum intercuspation

Patient number	Group I (Reference) *n* = 13	Group II (Test) *n* = 13
Mean ± SD (%)	8.08 ± 1.50	7.23 ± 1.59
95% CI	7.17, 8.98	6.27, 8.19
t Test (*p* value)	4.430 (0.001*)	

Group I, CAD-CAM zirconia crowns adjusted using buccal interocclusal record; Group II, CAD-CAM zirconia crowns adjusted using buccal and lateral interocclusal records. *Statistically significant difference at p value ≤ 0.05, t Test: Paired t test

According to Table 4’s Paired t test results (Fig. 12C), there was a significant difference in Group I’s mean crown volume values compared to Group II (p-value = 0.001).

**Table 4:** Comparison between groups regarding crown volume

Patient number	Group I (Reference) *n* = 13	Group II (Test) *n* = 13
Mean ± SD (mm^3^)	160.36 ± 15.94	157.63 ± 14.45
95% CI	150.73, 169.99	148.90, 166.36
t Test (*p* value)	4.291 (0.001*)	

Group I, CAD-CAM zirconia crowns adjusted using buccal interocclusal record; Group II, CAD-CAM zirconia crowns adjusted using buccal and lateral interocclusal records. *Statistically significant difference at p value ≤ 0.05, t Test: Paired t test

## Discussion

Most digitally designed prostheses are designed based on the MIP relationship, without taking the individual’s dynamic occlusion into account. Subsequently, longer time would be required for correction of occlusal interferences [25]. Modifying the prosthesis intraorally may alter the original anatomical shape created using the CAD software. For zirconia prostheses, such adjustments might induce tetragonal-to-monoclinic phase transformation, thereby significantly impact the mechanical characteristics [26].

Failure to eliminate the above-mentioned occlusal interferences even through intraoral modification could lead to various signs, including localized tooth ache, tooth mobility, occlusal wear and alterations in the masticatory chewing cycles [27, 28].

Additionally, it may be challenging to positively identify occlusal interferences intraorally due to various factors like poor vision, saliva and the limited marking capacity of articulating paper [29]. In this context, efforts should be made to minimize any potential occlusal errors during the occlusal design process.

Novel methods for incorporating individual functional movements into occlusal morphology have recently surfaced. These methods involve tracking mandibular motion using intraoral scanners. The acquired digital dataset is used to track mandibular movements and correct potential occlusal errors within the CAD software. This approach enables the Patient specific motion to reduce occlusal interferences during eccentric movements by accurately replicating functional mandibular motion in the CAD software [10].

A comparative analysis of three occlusal adjustment techniques revealed that only the digital device (T-scan) revealed occlusal interferences and occlusal discrepancy; articulating paper and intraoral 3D scanner did not reveal any occlusal deficiencies [30]. Comparative analyses have revealed a respectable degree of similarity in the T-scan and occlusence outcomes. In addition, Wan et al., [31] conducted research using four different tools to evaluate occlusal adjustments: articulating paper at 100 and 40 microns, Occlusense with a 60-micron thickness, and articulating silk at 80 microns. The findings indicated that the Occlusense digital device provided the most precise occlusal adjustments, followed by articulating silk, while the 40-micron articulating paper was found to be the least accurate.

The aim of this study was to propose a design methodology that incorporates the maximum intercuspal position (MIP) and the working-side lateral mandibular relation by acquiring an additional lateral interocclusal record and applying a suitable adjustment technique within CAD software.

The null hypothesis was refuted, since the crowns designed considering both patient’s static and dynamic occlusion displayed fewer occlusal interferences compared to those designed regarding static occlusion only. All crowns in this clinical study were fabricated using an identical zirconia blank, utilizing the same CAD-CAM milling machine and sintering furnace. Subsequently, both crowns for each patient were temporarily cemented using zinc oxide temporary cement before undergoing digital occlusal scheme analysis with Occlusense.

According to Park et al., [32] who introduced a digital technique for fabrication of restorations with consideration to lateral mandibular excursion, concluded that digital workflow had some limitations including that the interocclusal records for MIP and lateral excursion capture the initial and final points of mandibular movement towards the working side.

Therefore, it may be necessary to manually adjust the remaining portion, which typically results in lower levels of accuracy. Alternatively, obtaining additional interocclusal records between the maximum intercuspation position and the endpoint of the excursion can be considered to enhance the replication of lateral movement. Nevertheless, this alternative method frequently requires a significant amount of time and presents difficulties. The current study involved capturing a video recording of the lateral movement of the mandible for each participant. This recorded the entire path of motion, starting from the initial point (MIP) and ending at the final point of the movement. This approach eliminated any unguided adjustments and ensured more dependable outcomes.

In terms of the maximum pressure recorded during lateral mandibular movement on the working side, it was observed that Group I crowns exhibited a higher mean value compared to Group II crowns, and this disparity exhibited statistical significance. This discrepancy might be attributed to the elimination of the overlapping area between the designed crown CAD data (Group I) and the opposing arch scan data for lateral movement, resulting in the creation of statically and dynamically adjusted crowns in Group II.

These findings align with the results reported by Lee et al., [20] who investigated the treatment of single posterior zirconia crowns. They compared the occlusal surface following clinical adjustment with three different methods: no modification (design based on static occlusion), modification using the patient specific motion, and modification using a semi-adjustable articulator to assess occlusal errors. Occlusal errors decreased in a statistically significant way between the groups that received adjustments using PSM and the group that was designed using static occlusion. The occlusal discrepancies, particularly in cuspal inclines and triangular ridges, were notably decreased in posterior restoration fabricated considering patient specific motion.

Further evidence that this new digital workflow allows for the virtual removal of occlusal interferences is provided by Park et al.’s [32] study, which examined a digitalized work process for dental restorations considering lateral mandibular movement in CAD-CAM posterior crown design. The final restoration design exhibited a decrease in the cusp height and cusp inclination in comparison to the restoration designed utilizing standard workflow. These observations can offer contextual information for the outcomes of the existing study.

The present study identified a significantly higher mean pressure value recorded at maximum intercuspation in Group I crowns compared to Group II crowns. This observation could be attributed to the variations in cuspal inclination and height between the restoration designs generated using the conventional protocol and the new protocol.

Moreover, Li et al., [33] conducted a study on mandibular first molar crown design, examining wear facets following patient-specific motion capture using an intraoral scanner. They found that crowns designed with the patient-specific motion feature closely matched the original teeth’s wear facet morphology, surpassing those designed based on static occlusion and average-value virtual articulation. The study indicates that employing the patient-specific motion function may improve occlusal harmony in single restoration designs.

However, Attia et al.‘s [34] findings did not align with the outcomes of the present investigation. Their study, assessing the accuracy of restoring a missing first permanent molar using different occlusal recording techniques, involved three groups: Group (C) used a conventional mechanical articulator, Group (M) employed a mathematically simulated articulator, and Group (P) utilized patient-specific motion. Occlusense and articulating paper were employed to analyze occlusal accuracy. They determined that all three occlusal recording techniques yielded similar results with no significant differences.

Results of this study were also consistent with results obtained by Lin et al., [35], who used a digital occlusal analyzer (T-scan) to assess variations in the occlusion time (OT) and disocclusion time (DT) of individual posterior restorations featuring distinct designs. According to their findings, the functionally generated path (FGP) single crown significantly improved patient satisfaction and cut down on chairside occlusal adjustments time because it displayed remarkably less changes in OT and DT when compared to single restorations designed using conventional protocol. This was in agreement with the insight of the current study regarding the superiority of the design technique employing PSM over the conventional workflow.

Crown volume analysis revealed a significant difference between Group I crowns and Group II crowns. Group I had a higher mean crown volume. The removal of interferences from the occlusal surface of Group II crowns most likely impacted the calculations of crown surface area and volume.

The volume is computed by multiplying the crown surface area by the height that results from the center of mass, a measurement that the Meshmixer software assigns [24, 36]. The center of mass calculation produced nearly identical values, according to the results, but the surface area of Group II crowns was significantly smaller than that of Group I crowns. As a result of this decrease in surface area, there was a substantial reduction in the volume of the crown. Removing this substantial amount of tooth structure before the zirconia milling process would undeniably enhance the preservation of zirconia’s mechanical and aesthetic properties, as opposed to making corrections after the milling process. Furthermore, it would surely be beneficial for patient comfort and satisfaction to reduce chairside corrections.

According to de Carvalho et al.‘s [26] research, which examined the effects of aging, finishing and polishing methods on flexural strength of highly-translucent zirconia. Restoration adjustment with diamond burs had a negative effect on zirconia restorations’ fracture resistance as reported in the research. This is consistent with the viewpoint of the current study, which aims to prevent the need for additional finishing and polishing of the zirconia crowns after the milling process.

On the other hand, Pereira et al., [37] studied how diamond bur grinding affected the phase transformation and mechanical behavior of a Yttria-stabilized zirconia (Y-TZP) used in monolithic dental restorations. The study found that diamond bur grinding neither significantly impacted the ability of zirconia to maintain its integrity and strength under various mechanical stresses, nor affected the phase transformation of a Y-TZP ceramic.

This study is a clinical trial which makes it difficult to have perfect control on the entire variables. It focuses exclusively on comparing occlusal interference in single posterior crown restorations. Clinical studies utilizing alternative restoration position and lengths, such as a single anterior crowns or long-span fixed partial dentures, must be done to investigate the consequences of patient-specific motion (PSM) recording. The assessor in the present study was the provider, not dependent and blinded, which should be considered in future studies. Another limitation was the shape of the sensor on occlusal analysis device, which resembles the shape of the dental arch. Consequently, varying the positioning of the film sensor in the patient’s mouth would result in different recordings of the occlusal contact points’ locations, leading to significantly different values for the position of the center of contact forces or the sum of the percentage of forces in each quadrant.

The T-Scan, in contrast to the Occlusense, features a centering pin positioned in between the central incisors. However, even with the centering pin correctly attached to the maxillary dental arch, the sensor may still rotate around the midline, contributing to variability in the records. Consequently, the centering pin does not ensure a perfectly reproducible position of the piezoelectric film sensor. A recent study documented a new technique aiming to enhance the accuracy of recordings obtained with the two primary digital occlusal analysis devices available in the market, namely T-Scan and Occlusense. This method studied the use of the devices with a virtually designed customizable centering tray to ensure centering of the film sensor and thus more reliable digital occlusal analysis [38]. Further studies with higher sample size may be recommended.

## Conclusions

Based on the outcomes of this clinical study, the following conclusion was reached:

The digital functionally generated path technique allows for identifying occlusal interferences and modifying CAD-CAM zirconia crown designs.

## Data Availability

Data that support the findings of this study are available from the corresponding author, upon reasonable request.

## Abbreviations

3D: Three-dimensional
CAD-CAM: Computer-aided design and computer-aided manufacturing
DT: Disocclusion time
FGP: Functionally generated path
IOSs: Intraoral scanners
MIP: Maximal intercuspal position
OT: Occlusion time
PSM: Patient Specific Motion
STL: Standard tessellation language
Y-TZP: Yttria-stabilized zirconia
